# Evaluating the Efficacy of Antibiotic Therapy in Non-responsive Sacroiliitis: A Comparative Study

**DOI:** 10.7759/cureus.57372

**Published:** 2024-04-01

**Authors:** Ajay Bharti, Sanjay Kumar, Balram Omar, Shashank Prakash

**Affiliations:** 1 Orthopedics, All India Institute of Medical Sciences, Gorakhpur, Gorakhpur, IND; 2 Orthopedics and Trauma, Ganesh Shankar Vidyarthi Memorial (GSVM) Medical College, Kanpur, IND; 3 Microbiology, All India Institute of Medical Sciences, Rishikesh, Rishikesh, IND

**Keywords:** recovery rate, joa score, physical therapy, nsaids, antibiotics, sacroiliitis

## Abstract

Background: Sacroiliitis, characterized by inflammation of the sacroiliac joints, poses significant challenges in management, especially in patients unresponsive to standard therapies like non-steroidal anti-inflammatory drugs (NSAIDs) and physical therapy. This study aimed to evaluate the efficacy of antibiotic therapy in such patients, addressing a critical gap in the current treatment approach.

Methods: A total of 360 patients with lower back pain who presented to the outpatient department (OPD) of the Department of Orthopedics of a medical college in Northern India for six months were included in this study. With meticulous history taking, clinical examination, and radiological evaluation, 59 patients were diagnosed with sacroiliitis, out of which 31 were males and 28 were females, aged between 20 and 40 years, and were enrolled in this cross-sectional comparative study. Patients were divided into two groups: a control group (21 patients) receiving conventional treatment without antibiotics and a study group (38 patients) receiving conventional treatment plus antibiotics (who gave consent for treatment with antibiotics). The primary outcome was assessed using the Japanese Orthopaedic Association (JOA) score, with evaluations conducted at baseline, one month, and three months. Recovery rates were also calculated. SPSS trial software version 27 (IBM Corp., Armonk, NY) was used for statistical analysis.

Results: Both groups exhibited improvement in JOA scores over time. At the one-month and three-month follow-ups, the mean JOA scores and recovery rates showed no statistically significant difference between the control and study groups (p-values > 0.05). Adverse effects related to antibiotic use were not significant.

Conclusion: The study concludes that the addition of antibiotics to the conventional treatment regimen for sacroiliitis does not provide significant benefit in terms of functional recovery or pain relief in patients non-responsive to NSAIDs and/or physical therapy. These findings underscore the importance of a targeted treatment approach based on the specific etiology of sacroiliitis and caution against unnecessary antibiotic use.

## Introduction

Sacroiliitis, the inflammation of one or both sacroiliac joints where the spine meets the pelvis, has increasingly been recognized as a substantial cause of lower back pain [1]. This condition, often hidden behind more commonly diagnosed lumbar ailments, demands attention due to its diverse origins and clinical manifestations [2]. The complexity of sacroiliitis not only poses challenges in diagnosis but also complicates treatment approaches, often leading to protracted patient discomfort and recovery.

The etiology of sacroiliitis is multifaceted, with potential origins ranging from autoimmune to degenerative arthritis and traumatic injury to specific events like motor vehicle accidents or direct impacts to the buttock or pelvic area [3]. A particular demographic at risk includes women post-childbirth, where the stretching of the pelvis can lead to joint inflammation or ligament tears [4,5]. The subsequent excessive motion in these joints can foster degenerative changes and chronic pain. Moreover, the condition's severity is clinically graded from grade 0 (normal) to grade IV (complete ankylosis), indicating a spectrum from minor joint irregularities to complete fusion of the joint [1].

Traditionally, the treatment of sacroiliitis has focused on symptom alleviation, primarily utilizing non-steroidal anti-inflammatory drugs (NSAIDs) and physical therapy [6]. These interventions aim to reduce inflammation and manage pain while improving joint functionality. However, a subset of patients exhibits inadequate response to these standard therapies, prompting the exploration of alternative treatment modalities.

The role of antibiotics in treating sacroiliitis, particularly in cases not attributed to infection, has been an area of ongoing debate. The hypothesis driving the exploration of antibiotic therapy in non-infectious sacroiliitis cases posits that certain instances might involve undiagnosed or low-grade bacterial infections. This theory draws parallels from the success of antibiotics in treating chronic musculoskeletal conditions influenced by bacterial infections, such as chronic Lyme disease or reactive arthritis [7].

This study aims to rigorously evaluate the efficacy of antibiotic therapy in sacroiliitis patients who show poor or inadequate response to NSAIDs and physical therapy. By concentrating on this patient group, the research seeks to determine whether antibiotics can provide therapeutic benefits beyond their established role in infection management.

The significance of this study is manifold. First, it addresses a critical gap in current treatment strategies for sacroiliitis, especially for patients who are non-responsive to conventional therapies. Second, it explores the repurposing of antibiotics, an established drug class, potentially offering a cost-effective treatment alternative for sacroiliitis. Third, the study contributes to a broader understanding of the role of potential low-grade infections in chronic musculoskeletal disorders, which may influence future diagnostic and treatment strategies.

In terms of patient demographics, the study zeroes in on a younger age group, predominantly between 20 and 40 years. This focus is strategic, considering the higher physical activity levels and resultant susceptibility to functional impairments from sacroiliitis in this age bracket. Additionally, by excluding patients with secondary sacroiliitis stemming from conditions like degenerative arthritis, suspected cases of spondyloarthritis (SPA) trauma, or childbirth, the study ensures a focused examination of primarily sacroiliitis.

Anticipated results from this study could significantly influence clinical practice. If antibiotics are found effective, it could lead to a paradigm shift in sacroiliitis management and encourage further research into the underlying pathophysiology of the condition and the role of bacterial agents.

## Materials and methods

This cross-sectional study was designed to rigorously evaluate the efficacy of antibiotic therapy in the treatment of sacroiliitis, particularly in patients who have shown an inadequate response to conventional treatments like NSAIDs and physical therapy. The methodology employed in this study is detailed below, encompassing patient selection, treatment protocols, assessment tools, and statistical analysis.

Patient selection and grouping

The study included 360 patients with lower back pain who reported to the outpatient department (OPD) of the Department of Orthopedics at the Government Medical College and Hospital in Northern India for six months from December 2014 to June 2015. The patients who fulfilled the following criteria were included in the study: patients aged between 20 and 40 years with lower back pain, buttocks pain, and hip pain, with symptoms persisting for durations ranging from days to 12 weeks. Patients were excluded if their condition was associated with neurological symptoms, or radiculopathy, or was secondary to other causes such as degenerative arthritis, trauma, gynecological or obstetric conditions, post-childbirth complications, operative interventions, and HLAB27 positive, other bony or abdominal injuries, pathological fractures, or any infective etiology.

The diagnosis of sacroiliitis [1] was confirmed through a combination of patient history, clinical examination, and radiological investigations. Clinical examinations included local tenderness at the sacroiliac joint, Fortin's four-finger test, Flexion Abduction External Rotation (FABER) test, Gilly’s test, and Stork’s test. The patients were divided into two groups: (1) a control group (21 patients) managed conservatively without antibiotics and (2) a study group (38 patients) managed conservatively with antibiotics who consented to treatment with antibiotics.

Treatment protocol

The control group received conventional treatment [1] for sacroiliitis, which included NSAIDs, physical therapy, and lifestyle modifications as needed. The study group, in addition to the conventional treatment, was administered a course of antibiotics. The choice of antibiotics (fixed-dose combination: cloxacillin 500 mg + cefixime 200 mg + lactobacillus 90 million cells) was based on their broad-spectrum activity and tolerability. The antibiotics were given for two weeks [8] and standardized across the study group.

Assessment and follow-up

The primary assessment tool used in this study was the Japanese Orthopaedic Association (JOA) score [9,10]. This comprehensive score evaluates various aspects including pain intensity, joint functionality, and impact on activities of daily living. The JOA score was recorded for all patients at baseline (before starting the treatment) and then subsequently at one month and three months post-treatment initiation.

In addition to the JOA score, patient-reported outcomes and any adverse effects of the treatments were also monitored and recorded. Regular follow-up visits were scheduled to ensure adherence to the treatment protocol and to address any complications or concerns.

Statistical analysis

The data collected were subjected to thorough statistical analysis to evaluate the efficacy of antibiotic therapy in the treatment of sacroiliitis. The primary outcome measure was the change in the JOA score from the baseline to the follow-up points. The mean JOA scores were compared between the control and study groups using the Mann-Whitney U test, a non-parametric test ideal for comparing two independent samples.

The level of statistical significance was set at p < 0.05. Besides the primary outcome measures, secondary analyses included the assessment of the recovery rate and any adverse effects associated with the treatments.

Ethical considerations

This study followed the ethical guidelines laid down by the Institutional Review Board of the Ganesh Shankar Vidyarthi Memorial (GSVM)Medical College, Kanpur (IEC/RC/Ortho/02/ThesisMS/Oct2014). Informed consent was obtained from all participants, and they were assured of confidentiality and the right to withdraw from the study at any point without any consequences.

## Results

The results of the study focusing on the efficacy of antibiotics in the treatment of sacroiliitis in patients who are non-responsive to NSAIDs and/or physical therapy are presented below. The key findings relate to the changes in the JOA score and other relevant clinical outcomes.

Patient demographics

The study included 59 patients, with 21 in the control group (no antibiotics administered) and 38 in the study group (with antibiotics administered) as shown in Table 1. The demographic distribution was similar in both groups regarding age, sex, and occupation.

**Table 1:** Comparison of JOA score (baseline) between the groups based on antibiotic administration JOA: Japanese Orthopaedic Association.

Antibiotic administration	Number of patients (N)	Mean JOA score ± SD	Median JOA score
Not administered	21	22.38 ± 1.9	22.00
Administered	38	21.82 ± 1.4	22.00
Total	59	22.02 ± 1.6	22.00

Of these, 31 were male and 28 were female patients (Table 2).

**Table 2:** Distribution according to sex

Sex	Number	Percentage
Male	31	52.54
Female	28	47.45
Total	59	100.0

All patients were within the age range of 20-40 years, reflecting the study's targeted demographic (Table 3).

**Table 3:** Distribution according to age

Age (years)	Number	Percentage
20-30	19	32.20
30-40	40	67.80
Total	59	100.0

JOA scores at baseline

At baseline, the mean JOA score was 22.38 (SD ± 1.9) in the control group and 21.82 (SD ± 1.4) in the study group. The median JOA score was 22.00 for both groups, with ranges of 18.0-25.0 in the control group and 18.0-24.0 in the study group. This similarity in baseline scores allowed for a balanced comparison between the two groups. The Mann-Whitney test revealed no statistically significant difference between the groups (p = 0.2387) (Table 1).

JOA scores at one-month follow-up

At the one-month follow-up, the mean JOA score was 26.90 (SD ± 1.1) in the control group and 26.55 (SD ± 1.2) in the study group. The median score in both groups was 27.00, with a range of 24.0-29.0 in the control group and 23.0-29.0 in the study group. The Mann-Whitney test revealed no statistically significant difference between the groups (p = 0.2719) (Table 4).

**Table 4:** Comparison of JOA score (one month) between the groups based on antibiotic administration JOA: Japanese Orthopaedic Association.

Antibiotic administration	Number of patients (N)	Mean JOA score ± SD	Median JOA score
Not administered	21	26.90 ± 1.1	27.00
Administered	38	26.55 ± 1.2	27.00
Total	59	26.68 ± 1.2	27.00

JOA scores at three-month follow-up

At the three-month follow-up, the control group had a mean JOA score of 28.05 (SD ± 0.7), while the study group had a mean of 27.68 (SD ± 0.9). The median was 28.00 for both groups, with the control group ranging from 27.0 to 29.0 and the study group ranging from 24.0 to 29.0. Again, the Mann-Whitney test showed no significant difference between the groups (p = 0.1394).

**Table 5:** Comparison of JOA score (three months) between the groups based on antibiotic administration JOA: Japanese Orthopaedic Association.

Antibiotic administration	Number of patients (N)	Mean JOA score ± SD	Median JOA score
Not administered	21	28.05 ± 0.7	28.00
Administered	38	27.68 ± 0.9	28.00
Total	59	27.81 ± 0.9	28.00

Recovery rate (RR)

The RR at one month was 68.80% (SD ± 13.5) in the control group and 64.69% (SD ± 20.4) in the study group. The median was 71.43 for both groups, with the control group ranging from 71.43 to 100.0 and the study group ranging from 0.0 to 100.0. Again, the Mann-Whitney test showed no significant difference between the groups (p = 0.1394) (Table 6).

**Table 6:** Comparison of recovery rates (RR) at one month based on antibiotic administration JOA: Japanese Orthopaedic Association.

Antibiotic administration	Number of patients (N)	Mean JOA score ± SD	Median JOA score
Not administered	21	68.80 ± 13.5	71.43
Administered	38	64.69 ± 20.4	71.43
Total	59	66.15 ± 18.2	71.43

At three months, the RR improved to 85.23% (SD ± 9.8) in the control group and 81.50% (SD ± 14.7) in the study group. The median was 83.33 for both groups, with the control group ranging from 71.4 to 100.0 and the study group ranging from 16.7 to 100.0. The differences in RRs between the groups were not statistically significant at both one month (p = 0.6621) and three months (p = 0.7077) (Table 7).

**Table 7:** Comparison of recovery rates (RR) at three months based on antibiotic administration

Antibiotic administration	Number of patients (N)	Mean RR (%) ± SD	Median RR
Not administered	21	85.23 ± 9.8	83.33
Administered	38	81.50 ± 14.7	83.33
Total	59	82.83 ± 13.2	83.33

Adverse effects

**Figure 1:**
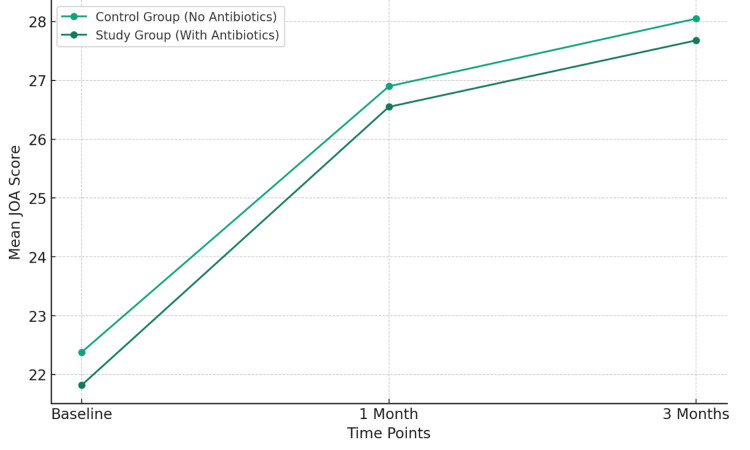
Comparison of JOA scores over time between the control and study groups JOA: Japanese Orthopaedic Association.

No significant adverse effects were reported in either group. The antibiotics were well-tolerated among patients in the study group. Figure 1 shows a line graph depicting the comparison of JOA scores over time between the control group (without antibiotics administration) and the study group (with antibiotics administration). The graph illustrates the mean JOA scores at baseline, one month, and three months for both groups.

From the graph, it is evident that both groups show a similar trend in the improvement of JOA scores over time. The scores increase from baseline to one month and continue to improve at three months, indicating an overall improvement in the condition of patients in both groups. However, there is no significant difference in the trajectory of improvement between the two groups, aligning with the study's conclusion that the addition of antibiotics does not significantly impact the recovery process in sacroiliitis patients who are non-responsive to NSAIDs and/or physical therapy.

## Discussion

The results of this study provide valuable insights into the role of antibiotics in the treatment of sacroiliitis, especially in patients who are non-responsive to standard therapies such as NSAIDs and physical therapy. Our study rigorously evaluated the efficacy of antibiotic therapy by comparing the JOA scores and RRs between a control group (without antibiotics administration) and a study group (with antibiotics administration). The findings indicate no significant difference in the improvement of JOA scores or RRs between the two groups over the one-month and three-month follow-up periods. Colmegna et al. [11] revealed that regardless of the causative organism(s), antibiotic treatment is generally ineffective.

Interpretation of results

JOA Scores Over Time

The similar trajectory of improvement in JOA scores in both groups suggests that the addition of antibiotics does not significantly enhance recovery in terms of pain relief, joint functionality, or quality of life. This lack of significant difference was consistent across the time points evaluated.

RRs

The RRs, which represent the percentage of improvement from the baseline, also did not show a significant difference between the groups. This further reinforces the notion that antibiotics do not significantly contribute to the overall recovery in sacroiliitis patients when added to conventional treatment regimens.

Implications for clinical practice

The findings from this study have important implications for clinical practice. First, they suggest that the prescription of antibiotics for sacroiliitis patients who do not respond to NSAIDs and physical therapy may not be beneficial. This is particularly relevant in the context of growing concerns about antibiotic resistance. Avoiding unnecessary use of antibiotics is critical in managing public health issues related to antibiotic overuse.

Second, the results underscore the importance of accurately identifying the etiology of sacroiliitis in each patient. Since the study's cohort did not include patients with confirmed infectious etiology, the lack of response to antibiotics in our study group suggests that non-infectious factors were likely predominant. Therefore, a more tailored approach to treatment, considering the specific underlying causes, may be more effective.

Limitations and future research

The study is not without its limitations. The sample size, though adequate for initial exploration, warrants larger-scale studies for more generalized conclusions. Additionally, the study's duration could be extended to observe the long-term effects of antibiotic therapy on sacroiliitis. Future research should also consider including patients with different etiologies of sacroiliitis to comprehensively understand the role of antibiotics across various subtypes of the condition.

Moreover, exploring the potential role of specific types of antibiotics, perhaps those with anti-inflammatory properties, might yield different results. It would also be beneficial to investigate the microbiological aspects of sacroiliitis more deeply, especially to identify any underlying low-grade infections that might not be apparent through standard diagnostic procedures.

## Conclusions

In conclusion, this study contributes significantly to the existing body of knowledge on the treatment of sacroiliitis. The results indicate that antibiotic therapy, when added to conventional treatment for sacroiliitis in patients who are non-responsive to NSAIDs and/or physical therapy, does not significantly improve outcomes in terms of JOA scores or RRs. This finding highlights the need for a more nuanced understanding of sacroiliitis, especially regarding its etiology and the development of targeted treatment strategies.
